# Enhanced immunogenicity of COVID-19 RBD fused to mutant scorpion's phospholipase D for vaccine development

**DOI:** 10.1016/j.nmni.2025.101634

**Published:** 2025-09-02

**Authors:** Mohammad-Reza Zareinejad, Mahdi Behdani, Mohammad Taghi Goodarzi, Akbar Oghalaie, Mohammad Hosseininejad-Chafi, Kamran Pooshang-Bagheri, Delavar Shahbazzadeh

**Affiliations:** a Department of Biochemistry, Shahrood Branch, Islamic Azad University, Shahrood, Iran; b Venom and Biotherapeutics Molecules Laboratory, Medical Biotechnology Department, Biotechnology Research Center, Pasteur Institute of Iran, Tehran, Iran

**Keywords:** Covid-19 vaccine, Receptor binding domain, Mutant phospholipase D, *Hemiscorpius lepturus* scorpion, Conventional virus neutralizing test, Vaccine

## Abstract

The management of the global COVID-19 pandemic relies, in part, on the rapid development of preventive vaccines at an unparalleled speed. The infection caused by the coronavirus is facilitated by the spike glycoprotein trimmer located on the surface of the virion, specifically through its receptor binding domain (RBD). The response of antibodies to this domain is a key indicator of the immunization's effectiveness and is well correlated with the neutralization of the viral agent. The failure of COVID-19 vaccines to induce complete neutralization provides an opportunity to find an alternative way. In this study, we show that recombinant RBD, Wuhan-specific; fused to mPLD1 protein, a highly effective immunogen from *Hemiscorpius lepturus* scorpion's venom, induce a potent immune response in mouse model. Conventional virus neutralization test (cVNT) with serum of mice immunized with mPLD1-RBD fusion protein, protects the Vero cells against the Omicron and Wuhan variants at the minimum of 1/64 and 1/32 dilution, respectively. The data strongly suggests that the subunit recombinant fusion protein could be a promising candidate for vaccine development against COVID-19. Our research showed that fusion of mPLD1, a highly effective immunogen, with RBD significantly boosts the immune response and substantially amplifies RBD antigenic properties in lab animals, setting the stage for their assessment in a comprehensive preclinical study.

## Highlights

• A novel recombinant Covid-19 vaccine uses a fusion protein combining the virus’s RBD domain with a mutated toxin derived from *H. lepturus* scorpion venom.
• sThis super-antigen significantly boosted antibody titers in animal models, effectively neutralizing the wild-type coronavirus.
• The innovative design also induced strong neutralizing responses against both Wuhan and Omicron variants.

## 1 Introduction

Coronavirus is a single-stranded positive-sense RNA virus, contains spike glycoproteins which extend from the viral surface, resembling a crown. The four key structural proteins of beta-coronaviruses include envelope protein (E), membrane protein (M), nucleocapsid protein (N), and spike protein (S). The receptor binding domain (RBD) of the viral spike (S) protein constitutes an optimal and efficacious target for vaccine development, given its critical role in mediating viral entry into host cells. Leveraging structural insights into the viral components and candidate proteins of coronavirus, it is anticipated that the entire S protein structure, including segments derived from the S1, S2, and RBD regions, will serve as primary target epitopes. These epitopes are pivotal in eliciting neutralizing antibodies against COVID-19 [[1], [2], [3], [4]]. Serological analyses of convalescent patients reveal the presence of neutralizing antibodies targeting various domains, particularly the RBD [5]. The primary strategy for controlling COVID-19 infection centers on the development of prophylactic vaccines. The initial attachment of viral particles is facilitated by the RBD of the spike (S)-glycoprotein trimmer, which binds to the host cell surface receptor, angiotensin-converting enzyme 2 (ACE2) [[6], [7], [8], [9], [10]]. Many COVID-19 vaccines are under development which aims to impede this process by targeting the entire S-protein or its RBD as antigens. The principal objective is to induce anti-RBD antibodies that disrupt the RBD-ACE2 interaction, thereby obstructing the initial step of infection [[11], [12], [13], [14], [15], [16]]. Virus neutralization depends on antibodies against the receptor binding motif (RBM) of RBD that directly interacts with ACE2. Key advantages of established recombinant subunit vaccine platforms include their robust safety profile, stability within the temperature range of 2–8 °C, and the ease with which production can be scaled up [17].

Immunization of animal models with folded RBD induces antibodies against conformational epitopes with high virus-neutralizing activity [18].

The recombinant RBD protein (26 kDa) typically exhibits weak immunogenicity and necessitates repeated vaccinations. This simplicity has led to subsequent evaluations in human subjects. However, most recombinant vaccines leverage macromolecular constructs which contain RBD to enhance immunogenicity, often incorporating potent adjuvant [18]. Fusion of hepatitis B virus (HBV) surface antigen (PreS) to RBD led to a considerable IgG response in rabbit model [18].

In our previous study, we showed that using a recombinant mutated Phospholipase D-1 (mPLD1) from the venom of *H. lepturus* scorpion could be a suitable alternative to the whole venom in producing horse antiserum [19]. In vivo tests of mPLD1, a 33 kDa protein, showed that the mice immunized with interval doses of 10 μg of mPLD1 completely protected against 10LD100 of the whole scorpion crude venom. They concluded that this mutant is an effective vaccine candidate against scorpion envenomation by *H. lepturus* in future clinical studies [19].

Here, we report the construction, expression, and purification of a COVID-19 subunit vaccine, mutant phospholipase-D1 (mPLD1) fused to RBD (mPLD1-RBD), and study of its efficacy to raise antibody response in vivo as well as its neutralizing efficiency against COVID-19 viruses, Wuhan and Omicron variants, in conventional virus neutralization assay.

The mPLD1 is a highly effective immunogen derived from the venom of a scorpion, *Hemiscorpius lepturus*, known for inducing protective immune responses as a promising adjuvant to development of fusion protein vaccines [19]. COVID-19 RBD domains are attached to the C-terminus of the mPLD1 protein. Therefore, it is anticipated that the immune response to the RBD protein will be significantly enhanced when fused with the mPLD1 protein.

## 2 Materials and methods

### 2.1 Reagent, bacteria, vector, and animals

PCR Master Mix was obtained from Viragen, Iran. DNA ligase, DNA ladder, *Xho*I, and *Nde*I were purchased from Thermo Fisher Scientific (USA). Pre-stained protein ladder and IPTG were purchased from Sinaclon, Iran. All chemicals and reagents were of analytical grade and obtained from Sigma Aldrich, USA. The TOP10 and BL21 (DE3) *E. coli* strains were employed for molecular cloning and the expression of the recombinant fusion protein, respectively. The pET-26b (+) vector (Novagen, USA) was utilized to clone the RBD protein gene downstream of the T7 promoter. All animal studies received approval from the Islamic Azad University, Shahrood Branch (IR.IAU.SHAHROOD.REC.1403.078). Prior to commencing their research, all researchers were acquainted with the ethical methods of handling laboratory animals. Vero cells was purchased from the cell bank of Pasteur Institute of Iran, Iran.

### 2.2 Construction of mPLD1-RBD gene

**Fig. 1:**
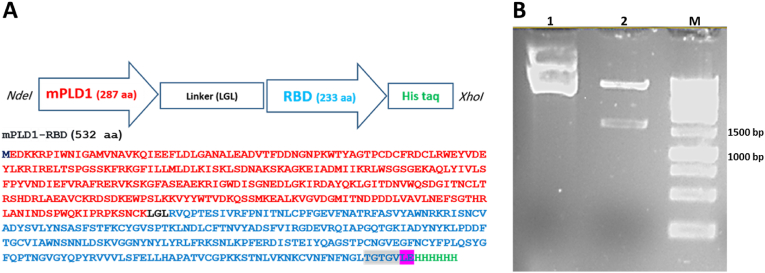
Construction of the mPLD1-RBD gene. A) Schematic representation of the gene construct for the fusion mPLD1-RBD protein and its amino acid sequences. Five amino acids at the C-terminal belonging to the S-protein are highlighted in gray, and two amino acids for the restriction enzyme sites are shown in purple. B**)** Gel electrophoresis of enzymatic digestion of the recombinant plasmid pET26b-mPLD1-RBD. Lane 1; undigested plasmid, Lane 2; digested recombinant plasmid, Lane M; DNA marker.

The mPLD1 and RBD gene constructs had been cloned and characterized [19,20]. The gene construct comprising the mutant phospholipase D (accession No.: KY287766.1) and the RBD coding sequence of the SARS-CoV-2 S protein (Wuhan-Hu-1, accession No.: YP_009724390.1, amino acids 319–546), together with the linker (LGL) and a His-tag at the C-terminus, sub-cloned into the pET26b vector. Briefly, the cloning method proceeded as follows: the mPLD1 gene was amplified using the T7 promoter and RBDpldF (ATCAAGCTTGGTCTGCGCGTTCAGCCGACCG) primers, which contains a *Hin*dIII cleavage site and an LGL linker at its terminus, and subsequently cloned into the pET26 vector between the *Nde*I and *Hin*dIII sites. Subsequently, the RBD gene was amplified with the T7 reverse and PLDrbdR (CTCAAGCTTGCAGTTAGATTTCGGACGCG) primers and cloned between the *Hin*dIII and *Xho*I sites (Fig. 1A). Enzyme digestion and sequencing were conducted for verification.

### 2.3 Expression of gene constructs

Initially, the mPDL-RBD, mPDL, and RBD gene constructs were individually transformed into BL21 bacteria. A confirmed colony of the transformed bacteria was then introduced into a culture medium containing kanamycin (100 μg/ml) and incubated overnight at room temperature. Finally, the expression was induced by IPTG at a final concentration of 0.5 mM at 28 °C overnight.

### 2.4 Purification of recombinant proteins

His-tag-mediated affinity chromatography was employed for purification using a Ni-NTA column (Qiagen, Germany). The IPTG-induced cells were harvested and re-suspended in phosphate-buffered saline (PBS 1 × , pH 7.4) containing NaCl (100 mM) and lyzed via sonication (20 s pulse, 10 s rest) for 10 min. The cell lysate was centrifuged at 10,000×*g*, at 4 °C, for 30 min. The pellets containing the inclusion bodies of RBD and mPLD1-RBD were resuspended in solubilizing buffer (NaCl 300 mM, Urea 8 M, NaH2PO4 50 mM, Imidazole 10 mM), incubated at 37 °C for 5 h in a shaker incubator, and sonicated again for 10 min. In order to LPS removal, the solubilized buffer was loaded on column and washing protocol was performed including 1 % Triton X-100. The column was equilibrated by solubilizing buffer which act as binding buffer too and the sample was loaded on and washed by this solution again. The bonded RBD and mPLD1-RBD were eluted by elution buffer (NaCl 300 mM, Urea 2 and 8 M, NaH2PO4 50 mM, Imidazole 500 mM). Subsequently, the purified proteins were refolded by refolding buffer (Urea 2 M) and dialyzed against PBS 1 × (pH 7).

Protein concentration was determined by the Bradford method. The recombinant protein was analyzed via 15 % SDS-polyacrylamide gel electrophoresis (SDS-PAGE). Finally, the expression was confirmed by Western blotting using anti-His tag antibody.

### 2.5 Potency assessment

Adult Balb/c mice (females, aged 6–8 weeks) with an approximate weight of 20–25 g were procured from the animal facility of the Pasteur Institute of Iran. All animals used in this study were cared for in accordance with the animal care and use protocol of the Pasteur Institute of Iran. Upon arrival, the mice were acclimatized for one week prior to conducting the experiments.

Groups of six mice were subcutaneously immunized six times at two-week intervals with 5, 5 and 10 μg of recombinant RBD, mPLD1 and mPLD1-RBD proteins, respectively adsorbed to complete Freund's adjuvant in the first injection, and to incomplete Freund's adjuvant in the subsequent injections. The groups received injections of proteins in a final volume of 100 μl. In this experiment, a group of mice injected with sterile PBS served as the negative control. After the last injections of mice, the blood collection was done.

### 2.6 Antibody assessments by ELISA and Western blotting

Blood samples were collected from the mice post-injection. The retro-orbital blood collection method used to collect the blood samples in mice. The collected blood volume is 0.5 ml using a pipette Pasteur at the timing of 15–20 s. The levels of antibodies against RBD, mPLD1, and the mPLD1-RBD fusion were assessed using ELISA which is coated by RBD, mPLD1, and mPLD1-RBD followed by blocking with skimmed milk. The sera obtained from immunized mice at a fix optimal dilution of 1:50 (100 μl), were added to the wells, and the plates incubated for 1 h at room temperature, followed by three washing steps with PBS. Subsequently, the plates were incubated with anti-mouse IgG HRP-conjugated (1:2000 dilution) at room temperature for 1 h, followed by three washing steps with PBS. The positive wells were identified by 100 μl of 3,3′,5,5′-tetramethylbenzidine (TMB) solution. The enzymatic reaction was terminated with 2N sulfuric acid, and absorbance was measured at 450 nm.

For Western blotting, mPLD1-RBD, mPLD1 and RBD proteins were electrophoresed on a 15 % SDS-PAGE gel and transferred onto nitrocellulose paper. Blocking was performed with 4 % skimmed milk at 4 °C for overnight. Following washing with PBS, the membrane was incubated with rabbit anti-His tag antibody at room temperature for 1 h. Following washing with PBS, the membrane was incubated with mouse HRP-conjugated anti-rabbit antibody at room temperature for 1 h. The band was visualized by adding 3,3′-diaminobenzidine (DAB) as a substrate.

### 2.7 Conventional virus neutralization test

The clinical isolates of SARS-CoV-2, including original Wuhan and Omicron strains, were isolated from nasopharyngeal swab samples, determined using the SARS-CoV-2 RT-PCR test (GA SARS-CoV-2 OneStep RT-PCR Kit), and confirmed via sequencing. All viral strains utilized in this study were subjected to complete genome sequencing. The resultant sequences have been deposited and are publicly accessible in the GISAID repository [ACCESSION NUMBER: EPI_ISL_7845314, ACCESS LINK: https://www.epicov.org/epi3/frontend#e66db].

Virus isolation and initial passage were carried out in Vero cells. SARS-CoV-2 was inoculated and titrated on Vero cells. Viral infectivity was calculated as tissue culture infectious dose 50 % (TCID50) via the Karber method. The conventional virus neutralization test (cVNT) was performed in a BSL3-certified reference laboratory (Amirabad Virology Lab, Tehran, Iran).

The test was performed on immunized mouse sera. Briefly, the collected serum samples were pooled and then decomplemented at 56 °C for 30 min. Heat-inactivated sera were mixed with wild type Omicron/Wuhan variants of COVID-19 in DMEM. Various serum dilutions were prepared, and 100 μl of each serum was mixed with 100 μl of virus (titer: 100 CCID50 per ml) for 1 h at 37 °C. The mixtures were then inoculated onto Vero cells in 96-well plates. Supernatants containing neutralizing antibodies were removed 1-h post-infection, and a semi-solid medium (agarose or carboxymethyl cellulose) was overlaid to prevent uncontrolled virus spread. Infected Vero cells were incubated in DMEM at 37 °C and 5 % CO_2_ for 72 h. Infected host cells lysed and infected neighboring cells, resulting in viral plaques observable by eye or microscope.

Typically, 4 days are required for COVID-19 to form visible plaques [21]. The number of plaque forming units (PFUs) was manually counted, assuming each plaque arose from a single infectious virus particle. Thus, PFU/ml indicates the number of infectious virus particles per ml, corresponding to viral infectivity [22]. If mice serum contained neutralizing antibodies (nAbs), cell infection was inhibited, reducing viral plaques. Higher nAbs titers correlated with fewer observed PFUs [23]. Neutralizing antibody titers were estimated as the highest dilution inhibiting CPE formation, as previously described [24]. Reference to the paper published by Gattinger P et al., in 2022, VNT titers ≥10 were considered positive.

### 2.8 Statistical analysis

Statistical analyses were performed by GraphPad PRISM software package, GraphPad Prism v10.4.2.633. The significance of differences between groups was assessed using ANOVA, with a significance threshold of *P* < 0.05.

## 3 Results

### 3.1 Gene construction

The sequence of RBD and mPLD1, fused by a linker (LGL), was cloned into the pET-26b expression vector, resulting in the recombinant plasmid, pET26b-mPLD1-RBD (NCBI accession number; PV550904) (Fig. 1A). Colony PCR was performed using T7 promoter and terminator primers, yielding a band of 1550 bp. The plasmid was then extracted and confirmed by enzymatic digestion with *Nde*I and *Xho*I restriction enzymes, revealing a 1550 bp band in the agarose gel (Fig. 1B).

### 3.2 Expression, purification and Western blot

**Fig. 2:**
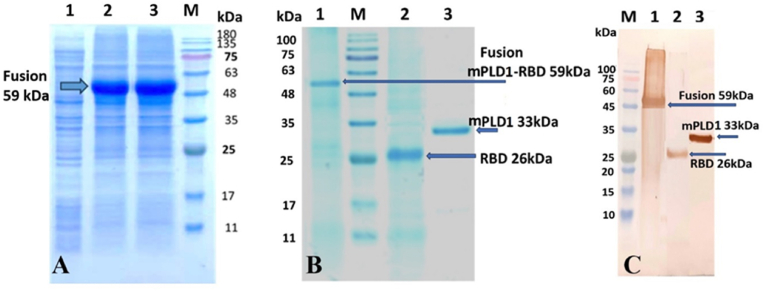
Characterization of recombinant mPLD1-RBD. **A**) Evaluation of gene expression by SDS-PAGE. Lane 1; negative control (before induction), Lanes 2 and 3; recombinant mPLD1-RBD after induction with 0.7 mM IPTG and incubation at 28 °C for 16 h, Lane M; protein marker. **B**) Recombinant proteins purification. Lane 1. mPLD1-RBD (2M Urea), Lane M, protein marker, Lane 2. RBD, Lane 3. mPLD1, **C**) Western blot analysis using anti-His antibody. Lane 1; mPLD1-RBD, Lane 3; mPLD1, Lane M; protein marker.

The highest expression was observed at 0.7 mM IPTG for 16 h incubation. The inclusion bodies containing RBD, and mPLD1-RBD fusion proteins were then dissolved and refolded. They were purified along with mPLD1 using a Ni-NTA column. SDS-PAGE analysis showed a fusion protein band at 59 kDa. Western blot analysis confirmed this with an anti-His antibody (Fig. 2).

### 3.3 Antibody raising against mPLD1-RBD

**Fig. 3:**
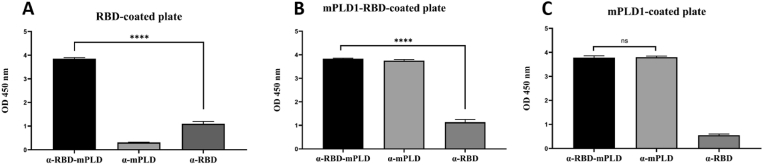
Reaction of recombinant antigen-coated plates with mice serum in ELISA test. A) RBD-coated plate. B) mPLD1-RBD-coated plate. C) mPLD1-coated plate. In all experiments, mice sera (α- RBD-mPLD1, α-mPLD1, and α-RBD protein) were collected after 6th injection. ∗∗∗∗P < 0.005.

Mice immunized with the recombinant mPLD1-RBD exhibited a significant increase in antibody levels (α-mPLD1-RBD) against RBD (Fig. 3A), mPLD1-RBD (Fig. 3B), and mPLD1 proteins (Fig. 3C) in the ELISA test while a weak antibody response (α-RBD) was observed against RBD in mice immunized with recombinant RBD (*P* ≤ 0.05) (Fig. 3A and B). These results indicate that RBD alone is insufficient to induce immunity in mice, but when fused to the mPLD1 protein, the immune response against RBD is significantly enhanced (*P* ≤ 0.05). No nonspecific antibodies were raised against mPLD1in the mice immunized against RBD (Fig. 3C).

### 3.4 Conventional virus neutralization test

**Fig. 4:**
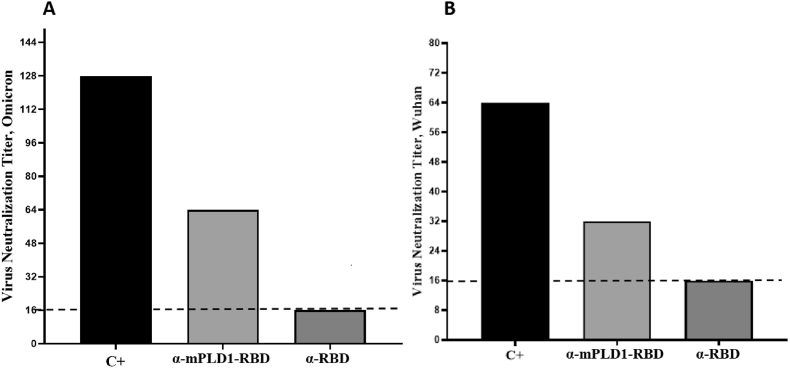
Neutralization of wild-type Omicron (A) and Wuhan (B) by α-mPLD1-RBD and α-RBD. The horizontal coordinate is dilution and the vertical is cVNT neutralization by mice serum. The dotted line denotes the detection threshold for neutralization in each experimental group.

Conventional virus-neutralizing activities were performed by using antisera derived from mPLD1-RBD immunized mice. The neutralizing activity of mice sera was evaluated with the COVID-19 conventional viruses. The virus growth curve was analyzed by CCID50. Serum of mice immunized with mPLD1-RBD fusion protein protected the Vero cells against the Omicron and Wuhan variants at the minimum of 1/64 and 1/32 dilutions, respectively (Fig. 4). As demonstrated, the serum of mice immunized with the mPDL1- RBD fusion protein exhibited a two-fold and four-fold higher neutralization capability compared to the serum of mice immunized with Wuhan and Omicron RBD alone, respectively.

## 4 Discussion

The first generation of COVID-19 vaccines introduced for global application predominantly comprised genetic vaccines and inactivated whole virus vaccines. However, there has been a notable increase in the development and application of subunit vaccines worldwide. Our design is based on the hypothesis that fusing the RBD of COVID-19 to a large carrier protein (mPLD1) can induce high titer of antibody against RBD. There are similar studies on the use of carrier proteins to enhance the immunogenicity of vaccines. Barkhordar et al. (2022) reported that coupling RBD to tetanus toxoid (TT) significantly enhanced both humoral and cellular immune responses [5]. SOBERANA-02, developed by the Cuban Finlay Institute is a recombinant RBD conjugated to TT [[25], [26], [27], [28], [29]]. Phase III clinical trials demonstrated 92 % efficacy (95 % CI: 86–95) against symptomatic COVID-19 and superior neutralizing antibody responses compared to conventional platforms [30].

Additionally, Gattinger et al. (2022) reported the construction and characterization of PreS-RBD, a COVID-19 subunit vaccine composed of two RBDs fused to the N- and C-terminus of PreS protein, the hepatitis B virus surface antigen. This design allows those unrelated proteins to work for each other as immunologic carriers. The interaction of RBD with ACE2 was more effectively inhibited by PreS-RBD-specific antibodies, and the concentration of virus-neutralizing antibody were higher than healthy individuals immunized with SARS-CoV-2 vaccines or COVID-19 convalescent cases [18].

Several studies demonstrated that unfolded RBD is not a promising immunogen so that no sufficient antibody is raised against it. Fusion of RBD with an immunogenic protein could have been resolved this issue. Our fusion protein is also capable of properly folding the RBD protein. Bellone et al. (2021) demonstrated that the RBD of the COVID-19, which is expressed in a prokaryotic host, *Escherichia coli*, was successfully refolded when fused to a fragment of diphtheria toxin (CRM197) [31]. Our study is consistent with the above mentioned study in terms of immunogenicity.

Gattinger et al. (2022) demonstrated that immunization with folded RBD induced raising of antibodies against conformational epitopes with high neutralizing activity [18]. In our study, the production of sufficient antibody in mouse serum to neutralize the COVID-19 virus in cVNT assay indicates the correct folding of the RBD protein. Our finding is in accordance with Gattinger et al. (2022) where *E. coli*-expressed folded PreS-RBD induced immune response rather than unfolded form.

As shown in Fig. 3, the high production of antibodies against RBD indicates that mPLD1 is a good candidate for enhancing presentation of much more RBD epitopes in antigen-presenting cells, suggesting further research in this area for use in non-responder patients against COVID-19 infection. Similar data observed by Sun et al. (2021) suggests that the RBD-Fc-based vaccine could induce broader neutralizing antibody than current vaccines [32].

Our previous in vivo experiments showed that PLD1 and mPLD1 are potent immunogenic proteins [19,33]. Accordingly, we used mutant PLD1 and fused it to RBD to enhance the ability of the new fusion protein to elicit an efficient immune response in a mouse model and demonstrated a significantly higher neutralizing antibody titer against Wuhan/Omicron in mice compared to naked RBD protein.

PLD exists in the venom of *H. lepturus* scorpion and brown spiders. *H. lepturus* is geographically distributed in Iran, Iraq, and other countries. Brown spiders, Loxosceles genus, is distributed in many countries of North and south America, including USA, Mexico, and Brazil, and other countries in this continent, also has phospholipase D as a major toxin. Concerning to the huge human population which lived in these countries, mPLD1 may be used for fusion with other protein vaccine like HBS antigen. In this case mPLD1 not only may increase the immunogenicity of HBS antigen but also anti-mPLD1 can be used for curing scorpion and spider envenomation. This issue is supported by the results of this study and our previous study in which the similarity of PLD in *H. lepturus* and Loxosceles genus has proved [33]. Furthermore, PLD is the safe vaccine candidate for prevention of envenomation in Iran and above-mentioned countries [19].

This study employed a fusion protein vaccination approach to enhance immunity. Mice immunized with the mPLD1-RBD fusion protein with Freund's adjuvant demonstrated the ability to develop high-titer antibody against RBD. Our previous study demonstrated no significant difference in the immune response of mice immunized with either Freund's adjuvant or alum [19]. The serum of the immunized mice exhibited promising neutralization capacity in cVNT assays against Wuhan and Omicron coronaviruses. Together, these findings demonstrate that immunizations of mice with the mPLD1-RBD protein induced raising of marginal levels of neutralizing antibodies against COVID-19 in the vaccinated mice.

## CRediT authorship contribution statement

**Mohammad-Reza Zareinejad:** Writing – original draft, Formal analysis, Data curation. **Mahdi Behdani:** Writing – review & editing, Supervision, Conceptualization. **Mohammad Taghi Goodarzi:** Writing – review & editing, Supervision, Conceptualization. **Akbar Oghalaie:** Investigation, Formal analysis. **Mohammad Hosseininejad-Chafi:** Investigation, Data curation. **Kamran Pooshang-Bagheri:** Writing – review & editing, Validation, Investigation. **Delavar Shahbazzadeh:** Writing – review & editing, Supervision.

## Informed consent statement:

Not applicable.

## Data availability statement

The original data is included in the article. Additional data can be provided upon request.

## Ethics approval

Animal experiments and protocols were conducted in accordance with the Ethics Committee of the Islamic Azad University, Shahrood Branch (IR.IAU.SHAHROOD.REC.1403.078).

## Funding

This work supported by Shahrood 10.13039/501100002660Islamic Azad University and 10.13039/501100010679Pasteur Institute of Iran, Grant No.: 1161 (Covid-19).

## Declaration of competing interest

The authors declare that they have no known competing financial interests or personal relationships that could have appeared to influence the work reported in this paper.
